# Testosterone Replacement Therapy: A Narrative Review with a Focus on New Oral Formulations

**DOI:** 10.17925/EE.2022.18.2.133

**Published:** 2022-08-24

**Authors:** Salman Z Bhat, Adrian S Dobs

**Affiliations:** Department of Endocrinology, Diabetes and Metabolism, Johns Hopkins Hospital, Baltimore, MD, USA

**Keywords:** JATENZO, male hypogonadism, oral testosterone replacement, self-emulsifying drug delivery systems, testosterone replacement therapy, testosterone undecanoate, TLANDO

## Abstract

Male hypogonadism affects 10–30% of the male population and is often under-recognized and under-treated. Different replacement formulations exist, each with specific benefits and limitations. These replacements include gels, patches and short- and long-acting injectables. JATENZO® (oral testosterone undecanoate; Clarus Therapeutics Inc., Northbrook, IL, US) is the first oral formulation of testosterone approved by the US Food and Drug Administration. TLANDO® (oral testosterone undecanoate; Lipocine Inc., Salt Lake City, UT, US), another oral testosterone formulation, has also recently been approved by the US Food and Drug Administration. Based on unique chemistry using a self-emulsifying drug delivery system and lymphatic absorption, JATENZO and TLANDO address some of the limitations of other dosing routes while providing a safe option without evidence of liver dysfunction. This review discusses various testosterone treatment options, focusing on the role and pharmacokinetics of the new oral formulations.

Depending on how it is defined, male hypogonadism affects between 10% and 30% of the male population and is often under-recognized and under-treated.^1–3^ It is defined by low sex hormone levels (<12 nmol/L or <300 ng/dL), which can affect multiple organ systems, resulting in symptoms and signs of testosterone deficiency (*Table 1*) and significantly reducing quality of life.^4–6^ Several studies have shown that patients with hypogonadism have an increased all-cause and cardiovascular mortality rate.^7,8^ It is also associated with increased risk of obesity and type 2 diabetes mellitus.^9–11^

Treatment with testosterone replacement therapy (TRT) is strongly indicated for the structural causes of hypogonadism (i.e. damage to the testes, pituitary gland or hypothalamus). Positive impacts of TRT on hypogonadism include improvements in erectile dysfunction, libido, lean body mass and bone mineral density.^12–14^ Beneficial effects on mood, cognition and metabolic parameters (body fat, insulin resistance and lipid levels) have also been noted.^15,16^ However, the impact of TRT on all-cause and cardiovascular mortality is controversial and not well defined.^17,18^ The benefit of TRT in age-related decline in testosterone is also not well established, and the US Food and Drug Administration (FDA) has discouraged treatment in this scenario.^19^

The US Endocrine Society and the European Academy of Andrology recommend testosterone levels in hypogonadal range (<12 nmol/L or <300 ng/dL) in three or more morning samples sent for biochemical analysis and symptomatic assessment (*Table 1*) before the diagnosis of hypogonadism.^20,21^ From the outset of diagnosis, the patient and healthcare provider should discuss and agree on the goals of therapy. Clinical conditions affecting testosterone-binding proteins (sex hormone binding globulin and albumin) result in falsely elevated/low total testosterone values, and the measurement of free testosterone is recommended in such scenarios (*Table 2*).^20,21^ Guidelines do not recommend universal screening for hypogonadism;^20,21^ however, physicians should remain more vigilant with patients at risk of hypogonadism (e.g. patients with HIV, pituitary disease or on chronic opioid therapy).

Caution is advised when treating age-related physiologic decrease in testosterone levels with TRT, as clinical data for the risks and benefits of TRT in the older patient population is not clear. The Testosterone Trials (The Testosterone Trials in Older Men; ClinicalTrials.gov identifier: NCT00799617) are a prospective analysis of TRT use in older men that showed moderate benefits with regards to sexual function (measured using the Psychosexual Daily Questionnaire-Q4 [PDQ-Q4] score for sexual activity, the Derogatis Inventory of Sexual Function-Men-II score for sexual desire and the International Index of Erectile Function [IIEF] score for erectile function) and a minor but significant effect on some aspects of mood symptoms (measured using the Positive and Negative Affect Score and the Patient Health Questionnaire-9 depression score).^22^ However, no significant benefit in primary outcomes of physical function and vitality were noted though.^22^ The trials were not powered to analyse risk.^22^ Ageing patients display a physiologic decline in testosterone levels, and treating these men in non-specific clinical scenarios (e.g. fatigue, decreased endurance) may do more harm than good.^19^ The Endocrine Society guidelines thus recommend an individualized treatment approach with a detailed discussion of risks in this patient population, even those patients with a hypogonadal biochemical and symptom profile.^21^

**Table 1:** Signs and symptoms of male hypogonadism

Symptoms of male hypogonadism	Signs of male hypogonadism
Sexual: decreased libido and erectile dysfunction, infertility Skin: reduced body hair growth, decreased frequency of shaving Psychiatric: low mood, decreased energy and motivation, mental fog, sleep abnormalities Fragility fractures Metabolic: increased adiposity, anaemia, features of metabolic syndrome	Loss of body hair Small testicular size (<5 cm) Gynaecomastia Low sperm count Abnormal sexual development in infants/children Musculoskeletal: decreased muscle mass, decreased bone mineral density

**Table 2:** Factors associated with increased (falsely elevated total testosterone) and decreased (falsely decreased total testosterone) sex hormone binding globulin levels

Increased serum SHBG concentration	Decreased serum SHBG concentration
Use of certain drugs (oestrogen, tamoxifen, phenytoin, barbiturates) Hyperthyroidism HIV Ageing Hepatocellular dysfunction (acute hepatitis, cirrhosis)	Use of certain drugs (androgens, progesterone, TNF-α inhibitors) Metabolic syndrome: obesity, non-alcoholic fatty liver disease Endocrine dysfunction (hypercortisolism, hypothyroidism, hyperprolactinaemia, acromegaly, type 2 diabetes mellitus) Pregnancy

*Measurement free testosterone concentration is helpful for ruling in/out hypogonadism in these clinical scenarios. HIV = human immunodeficiency virus; SHBG = sex hormone binding globulin; TNF = tumour necrosis factor.*

## Therapeutic options for male hypogonadism

Testosterone was artificially synthesized in 1935 by Butenandt and Ruzicka.^23^ Testosterone replacement therapy has since advanced from the early use of intramuscular testosterone propionate for ‘male climacteric’, as reported by Heller and Myers,^24^ to the gamut of dosing formulations currently available. The mode of administration should be primarily dependent on patient preference determined by comfort, ability and the side-effect profile. Bioavailability and improvement in plasma testosterone levels are the primary outcomes of the prospective trials that have studied these drugs.

TRT prescription rates have increased over the past 10 to 20 years, driven by claims of the energy- and endurance-boosting properties of testosterone. However, this increase might not be representative of the treatment of patients requiring therapy as per guidelines.^20,21^ Matching the right patient with the right drug formulation to ensure patient satisfaction and compliance is essential for maintaining long-term compliance. In fact, prescription refill rates have been as low as 25%.^25^ Therefore, individualizing therapy is important; fortunately, many options of TRT are available to achieve this goal (*Table 3*).

## Challenges of the existing formulations

Hypogonadism requires long-term treatment. Historically, patients have shown poor compliance to testosterone replacement therapies, displaying attrition rates of 30–90%, depending on the type of TRT and method of study.^26–28^ Ease of use, a better side-effect profile and the need for active laboratory follow-up/therapy adjustment are predictors of patient compliance to chronic therapy.

Injectable TRT is the most prescribed formulation in the USA healthcare market. Extended dosing periods and reliable physiologic effects are its main selling points (*Table 3*). Injections can be self-administered but are not an option for the needle-averse patient. Testosterone concentrations can become supraphysiological immediately after dosing and decline to sub-physiological levels at the end of a dosing cycle. This peak-and-trough cycle has significant physiological implications, with symptoms of elevated mood/anxiety at peak levels and fatigue, low mood and libido at nadir, before the next dose.^29^ Once injected, discontinuation of the injected drug in an acute setting is not possible due to its long duration of action. Pulmonary oil micro-embolism is a rare side effect associated with the use of ultra-long-acting intramuscular testosterone undecanoate (TU) (AVEED®; Endo Pharmaceuticals Inc, Malvern, PA, USA). It is clinically characterized by acute onset (<1 hour) of cough, likely resulting from the inadvertent injection of the oil formulation into the venous system.^30^

Topical testosterone therapy (gel/patch), although less invasive and easier to administer, can have variable absorption, risk of contact spread to females/children in the immediate family and contact allergies. Intranasal applications must be done thrice daily, which can be cumbersome and have nasal side effects. Subcutaneous implants have the benefit of chronic action, but placement requires a minimally invasive procedure and is not always convenient.

## Oral testosterone formulations: A new therapeutic avenue

The oral delivery route is the most common delivery system for the majority of medications. It solves many limitations of the alternative routes discussed above. Studies of other diseases have shown oral therapy to be preferred by patients compared with other routes.^31,32^ Oral testosterone was not a therapeutic option in the early stages of testosterone drug development due to its metabolism in the gut and high first-pass metabolism in the liver, which resulted in sub-therapeutic plasma levels.^33^ After 17 α-methylation was found to protect testosterone against hepatic metabolism, the 17-methyl-testosterone formulation had higher oral bioavailability and was approved by the FDA.^34^ However, the use of 17-methyl testosterone and other 17-α alkylated formulations is limited by the significant hepatotoxic effects noted in early case studies.^35–37^ The hepatotoxic effects include cholestasis, vascular injury (peliosis hepatis), transient hepatitis and hepatic tumour formation.^35–37^ The mechanism of hepatic injury is unclear, but it likely involves ductal and hepatocyte growth stimulus.

**Table 3:** Different formulations of testosterone replacement therapy

Route of administration (brand name)	Dosing	Advantages	Disadvantages
Buccal tablets (Striant®; Columbia Laboratories, Portland, OR, USA)	Twice daily, lymphatic absorption from buccal mucosa	Easy to use, discreet, easy to reverse	Twice daily dosing, gingival-related side effects, effects on taste, expensive
IM injection	Testosterone enanthate, tesosterone cypionate: 1–2 weeks; Testosterone undecanoate (AVEED®; Endo Pharmaceuticals Inc, Malvern, PA, USA): 10–12 weeks	Inexpensive generic, reduced dosing frequency, adjustable dosing, no transmissibility	Testosterone enanthate, tesosterone cypionate: Peak and trough levels leading to fluctuations in mood and libido, needle phobia, painful, skin irritation, higher risk of polycythaemia, need for dose adjustment, anaphylaxis. Testosterone undecanoate: POME and high cost (ultra-long acting)
Intranasal gel	Thrice daily	No transmissibility, not painful, easy reversibility, less impact on fertility?	Thrice daily dosing, nasal side effects (nasopharyngitis, sinusitis, epistaxis, parosmia)
Oral capsules: Andriol® (Merck Pharmaceuticals, Darmstadt, Germany), JATENZO® (Clarus Therapeutics Inc., Northbrook, IL, US), TLANDO® (Lipocine Inc., Salt Lake City, UT, USA)	Twice daily	Convenient, easy intake, no transmissibility, easy reversibility	Expensive, food-dependent absorption, gastrointestinal side effects (nausea, diarrhoea), elevated blood pressure
Subcutaneous implant/pellet (TESTOPEL®; Endo Pharmaceuticals Inc, Malvern, PA, USA)	Long acting (3–6 months)	Compliance, long-acting, no transmissibility	Invasive procedure, infection, skin allergies
Subcutaneous injection (XYOSTED™; Antares Pharma, Ewing, NJ, US)	Once weekly	Less painful than IM, no transmissibility, fewer fluctuations compared with IM	Expensive, elevated blood pressure
Transdermal patches	Once daily	Ease of use	Skin irritation, variable plasma concentration in some patients
Transdermal gels	Once daily	Ease of use	Skin irritation, transmissibility, daily dosing, variable plasma concentration in some patients

*IM = intramuscular; POME = pulmonary oil micro embolism.*

TU is an esterified form of testosterone introduced in Europe in the 1970s and is available in both oral and injectable formulations. The esterification of the 17 β position coupled with an oleic acid vehicle increased lymphatic absorption in the gut, thus bypassing the first-pass metabolism in the liver. A major advantage of TU is the considerably lower liver toxicity compared with the 17-methylated products in prospective studies. TU is partially metabolized in the intestinal wall, absorbed through intestinal lymphatics and converted primarily to 5-dihydro-testosterone. Intake with high-fat-content meals was important to aid absorption and achieve physiologic bioavailability. Earlier studies reported a peak serum testosterone level 2 to 6 hours after dosing, with high inter-individual variability.^38^ An oleic acid vehicle was used in the initial formulation, which needed to be kept refrigerated to maintain stability. In the early 2000s, it was replaced with castor oil/propylene glycol, which had equal efficacy but a better room temperature shelf life of 3 years.^39^ Oral TU in the above formulation has been approved for use in Europe, but it is not approved by the FDA due to erratic absorption, unsteady physiologic testosterone levels, patient-reported lack of efficacy and dependence on high dietary fat for absorption.^40^

**Figure 1:**
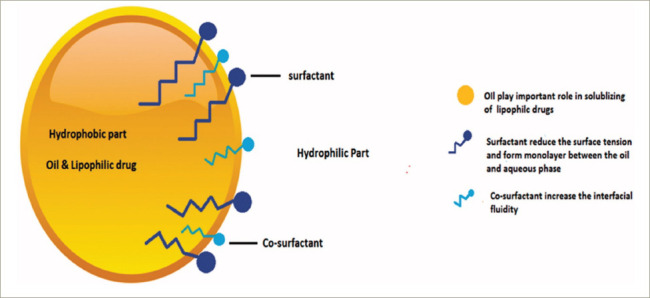
Components of self-emulsifying drug delivery systems

**Figure 2:**
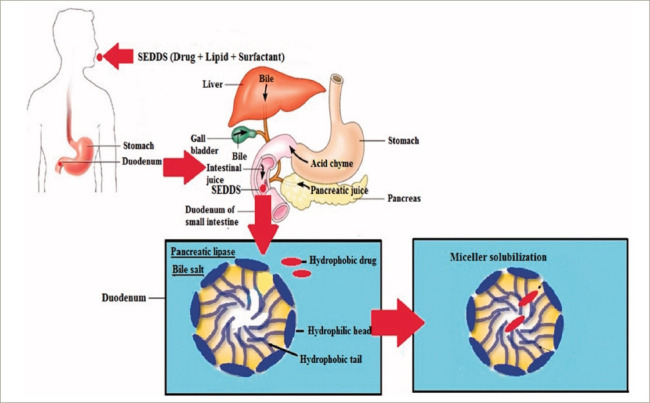
Micellar uptake of hydrophobic drug

**Figure 3:**
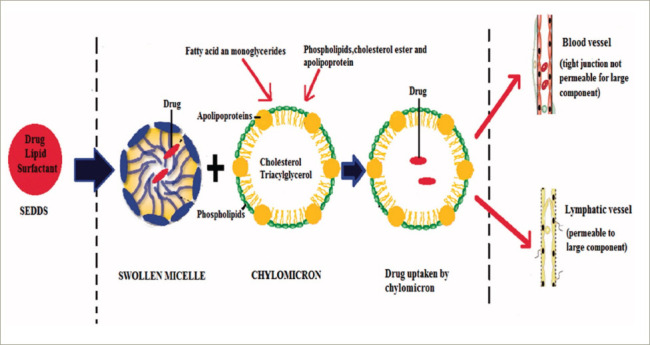
Lymphatic transportation of the hydrophobic drug in the lymphatic system

There had been an extensive search for a new drug delivery system that could provide reliable concentrations of testosterone (a hydrophobic molecule) and not require intake with a high-fat-content meal, which could place additional burden on therapy. The novel self-emulsifying drug delivery system (SEDDS) provided a breakthrough in achieving this.^41^ SEDDS consists of a surfactant with a high hydrophilic–lipophilic balance, a co-surfactant and a carrier oil dissolving the hydrophobic drug, which can self-emulsify in the gastrointestinal tract.^41^ Self-emulsification increases the surface area and enhances drug absorption in the lipophobic environment (*Figures 1–3*).^41^

**Figure 4:**
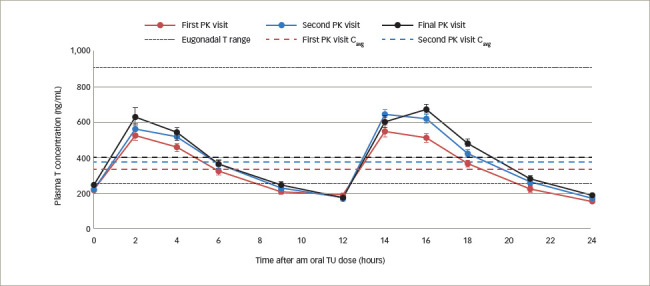
Plot of mean (± standard error) Na F-EDTA measured serum testosterone concentration versus time after dosing at the end of 24 days of therapy with twice-daily dosing of JATENZO in 151 subjects at different titration visits

Newer testosterone formulations based on SEDDS bypass the need for high-fat-content meals. In 2019, JATENZO® (TU; Clarus Therapeutics Inc., Northbrook, IL, USA) was the first oral TRT approved by the FDA for use in primary and central hypogonadism caused by structural and genetic defects. Its use for age-related hypogonadism has not yet been approved. It is available in different dosing formulations (158 mg twice daily [BID]; 198 mg BID; 237 mg BID), which helps in titration. The recommended starting dose is 237 mg BID. It still needs to be taken with meals but without the requirement of high-fat content. A phase III trial (A Study of Oral Testosterone Undecanoate [TU] in Hypogonadal Men [inTUne]; ClinicalTrials.gov identifier: NCT02722278) comparing JATENZO to topical testosterone (patients receiving JATENZO: n=166; oral:topical assignment: 3:1) reported comparable efficacy in improving testosterone levels to eugonadal range (87%) (24-hour bioavailability; *Figure 4*).^42^ Seventy-two percent of patients treated with the oral formulations required up-titration of the dose, which was performed twice. The titration process used average testosterone concentrations derived from 24-hour pharmacokinetic data on days 21 and 56 of the trial (average of the Na F-EDTA testosterone levels at 0, 2, 4, 6, 9 and 12 hours after the am dose and 0, 2, 4, 6, 9 and 12 hours after the pm dose). The 24-hour data correlated very well with single-point testosterone concentrations (between hours 4 and 6 after oral TU administration), which is typically used in the outpatient setting. The testosterone levels achieved did not depend on the fat composition of meals.

*Reproduced with permission from Taylor & Francis Ltd. from Chatterjee B, et al (2016).^41^*

*Reproduced with permission from Taylor & Francis Ltd. from Chatterjee B, et al (2016).^41^ SEDDS = self-emulsifying drug delivery system.*

*Reproduced with permission from Taylor & Francis Ltd. from Chatterjee B, et al (2016).^41^ SEDDS = self-emulsifying drug delivery system.*

*Eugonadal testosterone range: 300–1000 ng/dL. Source: Swerdloff et al. (2020).^42^*

*C_avg_ = average testosterone concentrations; PK = pharmacokinetics; T = testosterone; TU = testosterone undecanoate.*

JATENZO led to statistically significant improvement in sexual and mood symptoms (measured usign the PDQ), comparable to topical TRT. Common side effects included gastrointestinal reactions (nausea, diarrhoea), which occurred at significantly higher rates in the oral TRT group. Besides the previously defined class side effects, JATENZO has a small but significant increase in 24-hour average systolic blood pressure (JATENZO 4.9 ± 8.7 mmHg versus topical testosterone 0.2 ± 9.4 mmHg; p=0.0013) along with an increase in low-density lipoprotein cholesterol and a decrease in high-density lipoprotein cholesterol. No increase in the incidence of liver dysfunction was noted. JATENZO was also found to significantly improve quality of life (measured by improvement in the 36-Item Short Form Survey and PDQ, p<0.0001), bone mineral density (mean increase in bone mineral density over 180 and 365 days in the spine was 0.013 ± 0.035 and 0.018 ± 0.042 g/cm^2^, respectively, and in the hip 0.006 ± 0.019 and 0.012 ± 0.023 g/cm^2^, respectively; p<0.0001) and lean body mass (an increase of 2.87 ± 2.73 and 3.15 ± 2.69 kg at 180 and 365 days, respectively, (p<0.0001) compared with baseline.^43^

**Figure 5:**
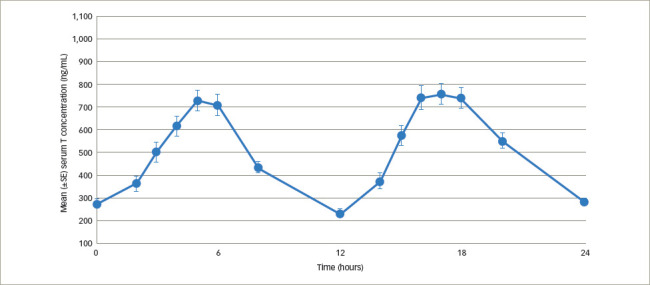
Plot of mean (± standard error) serum testosterone concentration versus time after dosing at the end of 24 days of therapy with fixed dose 225 mg twice daily of TLANDO in 90 subjects

*Source: DelConte et al. (2022).^47^*

*SE = standard error; T = testosterone.*

TLANDO® (Lipocine Inc., Salt Lake City, UT, USA), another oral formulation of TU using SEDDS, has recently been approved by the FDA for the treatment of male hypogonadism.^44^ As this formulation does not require dose titration, patients do not need to have regular blood work, making use and follow-up easier. A 52-week open-label multicentre study in men diagnosed with hypogonadism comparing a titratable regimen of TLANDO (n=210) to topical testosterone (n=105) showed similar average and maximum concentrations of testosterone, with an equal proportion of study participants achieving eugonadal levels.^45^ Beneficial effects were noted in sexual and mental domain patient-reported outcomes.^46^ The effect of titration on the achieved testosterone levels was minimal. Subsequently, an open-label, single-arm trial studying the efficacy and safety of a fixed dose of TLANDO (225 mg BID) treatment for 24 days on hypogonadal men (N=95) demonstrated steady 24-hour average serum testosterone levels in 80% of patients without the need for titration (Dosing validation study of oral testosterone undecanoate [TU, LPCN 1021]. [DV]; ClinicalTrials.gov identifier: NCT03242590).^47^ *Figure 5* shows the 24-hour bioavailability of TLANDO. The effect of fat content in food was studied in a smaller cohort and did not show any difference in bioavailability between different diets. Another trial evaluating a fixed dose regimen of 150 mg thrice daily did not meet efficacy targets.^48^ Intake with meals is necessary for absorption; however, no requirement of fat content is necessary.

A single-arm study to evaluate the effect of TLANDO on different blood pressure parameters (participants who received at least 1 dose of the study drug: n=138) noted a mean increase of 3.8 mmHg (95% confidence interval: 1.7–6.0) and 1.2 mmHg (95% confidence interval: 0.3–2.1) in 24-hour ambulatory systolic and diastolic blood pressure, respectively.^49^ The mean increase was higher in patients with baseline hypertension compared with non-hypertensive subjects (4.5 mmHg/1.5 mmHg versus 3.2 mmHg/0.9 mmHg, respectively).^49^ The increase in ambulatory blood pressure was highest in the quartile with the highest haematocrit increase, promoting the hypothesis of haematocrit-induced hypertension.

Interestingly, a prodrug of TLANDO, LPCN 1144 (ipocine, Inc., Salt Lake City, UT, USA) was also noted to decrease the hepatic fat content in a subset of patients from the ambulatory blood pressure trial, and its benefit in treating non-alcoholic fatty liver disease, which is associated with male hypogonadism, is currently being studied.^50^

Another novel oral TU option (KYZATREX™; Marius Pharmaceuticals, Raleigh, NC, USA) that uses a phytosterol carrier vehicle is being studied in the RE-TUne study (Efficacy and safety of oral testosterone undecanoate in hypogonadal men; ClinicalTrials.gov identifier: NCT03198728), but results have not been published yet.^51^

## The hypertensive effect: Route or class specific?

Previous trials using TRT for hypogonadism did not address the cardiovascular risk with adequate power. The analysis of cardiovascular outcomes from epidemiological, prescription data and retrospective studies have shown conflicting results. Among others, the results of the prospective Testosterone in Older Men with Mobility Limitations (TOM: Testosterone in Older Men With Sarcopenia; ClinicalTrials.gov identifier: NCT00240981) trial^52^ and the retrospective study of hypogonadal men who underwent coronary angiography, subsequently treated with TRT,^17^ among others, demonstrated a significant increase in major adverse cardiovascular events. As a result, there is an increased focus on evaluating these outcomes in future TRT formulations.

Most of the side effects reported for oral TRT represent a class effect, including headache, elevated prostate-specific antigen, increased haematocrit and a change in serum lipids. A significant elevation in blood pressure in the oral TRT arm compared with the control arm merits further discussion. Both TLANDO and JATENZO and a new self-injectable medication, testosterone enanthate (XYOSTED®; Antares Pharma, Ewing, NJ, USA) have shown elevations in ambulatory blood pressure compared with the treatment arm. JATENZO is also associated with increased low-density lipoprotein cholesterol levels, whereas TLANDO is not.

**Table 4:** Benefits and limitations of oral testosterone replacement therapy

Benefits	Limitations
Easy administration Easy storage Low risk of secondary exposure Steady serum testosterone levels	Expensive/Limited insurance coverage Dyslipidaemia and hypertension GI side effects Need for intake with food

*GI = gastrointestinal.*

Hypertension is a major cardiovascular risk factor, and, given the unclear association of TRT with increased cardiovascular disease, worsening blood pressure may be detrimental to the long-term use of TRT. Possible mechanisms suggested include a rise in haematocrit and sodium and water retention. It is unclear why oral TRT might be the only therapy with this side effect. Erythrocytosis is a class-wide effect of TRT. TRT stimulates erythropoietin release, increases iron absorption by decreasing hepcidin levels and directly enhances erythropoiesis.^53^ An increase in haematocrit, seen with both JATENZO and TLANDO, has been strongly associated with an increase in blood pressure.^54,55^ Elevation in blood pressure from TRT is likely to be a class-specific side effect, which was not demonstrated with other routes as earlier studies had not specifically looked at outcomes of blood pressure with ambulatory monitoring techniques, which are more sensitive for capturing blood pressure changes.

## Conclusion

Oral TRT is an important advance in testosterone replacement options for patients requiring treatment. Oral replacement provides ease of administration with reliable serum levels and adequate efficacy (*Table 4*). The FDA-approved oral TU formulations discussed above provide reliable testosterone levels when compared with topical TRT, without the need for ingestion with a high-fat-content meal and with no additional incidence of hepatotoxicity. The clinical benefit has also been demonstrated in these studies, although it has not been their primary outcome. Patient satisfaction is similar to other forms of TRT, compared with previous oral TRT medications that were often associated with a decreased efficacy by patients.^56^ Long-term safety and efficacy data are not available, and direct comparisons to other forms of TRT have not been performed yet. The oral formulations have shown small but significant elevations in blood pressure during ambulatory blood pressure monitoring, which is likely a class-specific effect. With the appropriate monitoring of serum testosterone levels, blood pressure and complete blood count, oral testosterone is another option for men requiring testosterone treatment.
